# The multichromosomal structure evolution of *Dendrobium* mitogenomes and new insights into interrelationships of recently radiated tribes in Epidendroideae (Orchidaceae)

**DOI:** 10.3389/fpls.2026.1864920

**Published:** 2026-06-05

**Authors:** Mengting Wang, Jiawei Jiang, Hongdou Liang, Lijia Lei, Yalu Chen, Yinshuai Li, Yunyan Hua, Tao Ma, Wona Ding, Xu Li, Zhitao Niu

**Affiliations:** 1 Ningbo Key Laboratory of Agricultural Germplasm Resources Mining and Environmental Regulation, College of Science and Technology, Ningbo University, Cixi, China; 2 School of Design, Shanghai Jiao Tong University, Shanghai, China; 3 College of Life Sciences, Key Laboratory of State Forestry and Grassland Administration for Dendrobium Officinale, College of Life Sciences, Nanjing Normal University, Nanjing, China

**Keywords:** *Dendrobium*, Epidendroideae, mitochondrial genome, multi-chromosomal structure, Orchidaceae, taxonomy

## Abstract

**Introduction:**

Epidendroideae is the largest subfamily in Orchidaceae, comprising over 20,000 species in 16 tribes, which are renowned for their remarkable morphological diversity and beautiful flowers. Although phylogenetic relationships have been well-resolved in most tribes of Epidendroideae, controversies remain among some recently radiated tribes. To advance the knowledge of Epidendroideae relationships, we used complete mitochondrial sequences to reconstruct the phylogeny of 10 orchid species from the six recently radiated tribes of Epidendroideae.

**Methods:**

We first *de novo* assembled the complete mitogenome of *Dendrobium chrysanthum* using Illumina and Nanopore data. A comparative analysis was then conducted on nine mitogenomes from three orchid subfamilies. Additionally, we newly assembled 10 mitochondrial genomes of orchid species from six Epidendroideae tribes for phylogeny reconstruction and reconstructed both the mitochondrial and plastid phylogenies for these tribes.

**Results and discussion:**

The mitogenome of *D. chrysanthumwas* was 638,041 bp long with a multichromosomal structure. The comparative analysis of nine orchid mitogenomes revealed great variations in the structure and genome size, while sequence similarities among these mitogenomes were closely related to the phylogenetic affinity. Most clades were resolved in mitochondrial and plastid phylogenies. Moreover, the comparative analysis of phylogenetic topology showed discordances between the plastid and mitochondrial phylogenies. Our findings enriched the mitogenome database and the knowledge of evolutionary features of orchid mitogenomes. The whole genomic sequences of mitogenomes provided new insights into the interrelationships of Epidendroideae (Orchidaceae).

## 1 Introduction

The Orchidaceae (Orchids) represent one of the largest families of flowering plants, comprising approximately 27,000 species across more than 750 genera, occurring in tropical and subtropical regions of every continent except Antarctica (POWO, 2024). Orchid species are renowned for their remarkable morphological diversity, characterized by numerous intergrades and overlapping traits (Adams, 2011). The significant morphological divergence among species posed challenges for taxonomy. Some early studies for orchid classification based on morphological data were inconsistent with one another (Bateman et al., 2005; Chase et al., 2015). A key point of contention revolves around the evolution of stamen numbers (Vermeulen, 1966; Dahlgren et al., 1985; Szlachetko, 1995). In earlier classifications, flowers with a single stamen were thought to be more morphologically advanced than those with two or three stamens (Cameron et al., 1999). Recent molecular phylogenetic analyses confirmed the classification of orchids into five subfamilies, including Epidendroideae, Orchidoideae, Apostasioideae, Cypripedioideae, and Vanilloideae (Chase et al., 2003). The phylogeny of complete plastome sequences revealed that Epidendroideae and Orchidoideae were sister groups to the Cypripedioideae (multi-stamened subfamily), rather than to the Vanilloideae (one-stamened subfamily) (Niu et al., 2017).

As the largest of the five subfamilies, Epidendroideae comprises more than twenty thousand species (approximately 76% of the orchid family) according to the Catalogue of Life (https://www.catalogueoflife.org/). Although significant progress has been made in resolving the phylogenetic relationships within orchids, controversies remain, particularly among some recently radiated tribes, especially within the Epidendroideae subfamily (Zhang et al., 2015; Li et al., 2019; Serna-Sanchez et al., 2021; Perez-Escobar et al., 2024; Zhang et al., 2023). The relationships of three Epidendroideae tribes (Cymbidieae, Epidendreae, and Vandeae) showed inconsistencies in different studies. The phylogeny of 39 orchid species based on 75 chloroplast genes revealed that the Cymbidieae clade and Vandeae clade were clustered into monophyletic group, and the Epidendreae clade was paraphyletic with these two clades (Givnish et al., 2015). The results of Wong and Peakall (2022) also supported the monophyly relationship between the Cymbidieae clade and Vandeae clade. However, the reconstructed tree using the Orchidaceae963 gene set strongly supported that Cymbidieae and Epidendreae were monophyly, which was a sister clade to Vandeae (Eserman et al., 2021). Besides, the plastid phylogeny analyses of 74 orchid species from 18 tribes also showed monophyly relationships of Cymbideae and Epidendreae (Li et al., 2019). Different sampling strategies and DNA markers played a significant role in such inconsistencies of Epidendroideae tribe relationships.

Plant mitochondrial genomes are remarkably large and structurally complex compared to those of animals, ranging from 66 kb (*Viscum scurruloideum*) to 11.3 Mb (*Silene conica*) in size (Skippington et al., 2015; Sloan et al., 2012). They exhibit extensive variation in genome structure, including frequent rearrangements and the presence of large repeat sequences that mediate recombination (Sullivan et al., 2020). This high level of structural dynamism contributes to the diversity and evolution of plant mitochondrial genomes across species. Benefiting from developed sequencing technology, a growing number of plant mitochondrial genomes have been sequenced, yet they remain underrepresented compared to nuclear and chloroplast genomes (Kozik et al., 2019; Liu et al., 2023; Zhou et al., 2023; Yang and Duan, 2024). Currently, fewer than thirty mitochondrial genomes have been published for Orchidaceae species according to NCBI (https://www.ncbi.nlm.nih.gov/). Despite such limitations, mitochondrial genomes have proven valuable in phylogenetic studies, particularly for resolving deep evolutionary relationships due to their conserved sequences (Horn et al., 2017; McCauley, 2013; Pearl et al., 2009). For instance, phylogenetic relationships of 26 *Dendrobium* species were successfully reconstructed using mitochondrial genomic data (Wang et al., 2023). Besides, the 38 mitochondrial protein-coding genes were used to resolve relationships of 74 orchid species from 18 tribes, and the relationships of most tribes and subtribes were well resolved (Li et al., 2019). Mitochondrial phylogenies often provide new insights into relationships in many plant lineages compared with plastid and nuclear phylogenies (Wang et al., 2022; Richardson et al., 2013). However, mitochondrial phylogenetic relationships of several recently radiated tribes within Epidendroideae were still unsolved, such as Vandeae, Collabieae, and Cymbidieae, which needed more mitochondrial sequences to resolve (Givnish et al., 2015; Perez-Escobar et al., 2024).

To expand the mitochondrial genome database of Orchidaceae and resolve phylogenetic relationships of several recently radiated tribes within Epidendroideae, we assembled mitochondrial genomes of ten orchid species representing six tribes within Epidendroideae, compared genomic diversity of nine orchid mitogenomes, and reconstructed the mitochondrial phylogeny of these orchid species.

## 2 Materials and methods

### 2.1 Taxon sampling

To reconstruct the interrelationships of six recently radiated tribes in Epidendroideae, we collected 10 orchid species from these tribes, including Vandeae, Collabieae, Cymbidieae, Epidendreae, Podochileae, and Malaxideae (**Supplementary Table 1**). Two species (*Vanilla annamica* and *Allium cepa*) were selected as the ingroup and outgroup, respectively. The root tips of *Dendrobium chrysanthum* (Epidendroideae, Malaxideae) were sampled from tissue culture seedlings that were cultivated on the MS medium (Lin et al., 2020). All accessions were identified by Prof. Zhitao Niu and stored at the greenhouse of Ningbo University.

### 2.2 DNA extraction and sequencing

The mitochondria were extracted from 5 g of root tips of *Dendrobium chrysanthum* (Orchidaceae, Epidendroideae) through optimized centrifugation techniques (Gui et al., 2016). Mitochondrial DNA (mtDNA) was isolated from purified mitochondria utilizing a modified SDS protocol (Chen et al., 2011). The certified mtDNA samples (concentration ≥ 20 ng/μl, A260/230 > 1.7, A260/280 = 1.8-2.0) were chosen for library preparation, including a 10 kb library for Nanopore sequencing and a 150 bp library for Illumina sequencing, both using the ONT kit (SQK-LSK114). The Nanopore library was sequenced on the PromethION platform (Oxford Nanopore Biosciences, Cambridge, USA) using the R10.4.1 chip, yielding approximately 10 Gb long-reads. For short-read sequencing, the Illumina library was loaded onto the Hiseq4000 platform (Illumina, San Diego, USA). We obtained approximately 10 Gb raw data from the Hiseq4000 platform. The long-read sequencing data were subsequently corrected using the short-read sequencing data with LoRDEC (k-mer value = 19; abundance threshold = 3) (Salmela and Rivals, 2014).

For total DNA extraction, approximately 0.2 g of fresh leaves was collected from 10 orchid samples (**Supplementary Table 1**) and two ingroup/outgroup samples (*Vanilla annamica* and *Allium cepa*). Total DNA was isolated using the DNeasy Plant Mini Kit (Qiagen, Hilden, Germany). The certified total DNA samples were sequenced on the Illumina Hiseq4000 platform (Illumina, San Diego, USA), generating 5–7 Gb paired-end reads. Low-quality reads were excluded from the raw data using CLC Genomics Workbench 8.5.1 (CLC Biosciences, Aarhus, DK).

### 2.3 Mitogenome assembly, and annotation

Two strategies were used to assemble mitochondrial genomes (Wang et al., 2023). The assembly of the *D*. *chrysanthum* mitogenome was performed using a *de novo* strategy implemented in SPAdes v3.10.1 using mtDNA sequence data, including long-read and short-read data (k-mer values: 21, 33, 55, 77, 99; phred-offset: 33) (Bankevich et al., 2012). The assembled scaffolds with more than 20-fold coverage (calculated by Illumina reads) were further blasted against the local mitochondrial genome database using BLASTN (**Supplementary Table 2**). Then, we obtained mitochondrial-related scaffolds and polished these scaffolds by BWA and Pilon to correct any erroneous bases or indels (Li and Durbin, 2010; Walker et al., 2014). For phylogenetic analyses, we used the mitogenome of *Dendrobium huoshanense* (LC657527- LC657545) as the reference to assemble the mitogenomes of 10 orchid samples (**Supplementary Table 1**) and two ingroup/outgroup samples (*Vanilla annamica* and *Allium cepa*) based on Genomics Workbench 8.5.1, using total DNA sequence data. To avoid chloroplast-derived sequences interfering with the phylogenetic relationships, we removed these chloroplast-derived regions from the reference mitogenome (Wang et al., 2023). A custom database consisting of mitochondrial genes from 48 angiosperms was applied for the annotation of mitochondrial genomes using BLASTN (e-value < 1e−5) (**Supplementary Table 2**). We also manually verified and corrected the exon boundaries, as well as start and stop codons of protein-coding genes, using Vector NTI (Lu and Moriyama, 2004). The tRNA genes were annotated through tRNAscan-SE version 1.21 (Schattner et al., 2005).

### 2.4 Chloroplast genome assembly

The chloroplast genomes of six orchid species (*Phalaenopsis amabilis*, *Holcoglossum flavescens*, *Maxillaria tenuifolia*, *Trichotosia dasyphylla*, *Pinalia spicata*, and *Vanilla annamica*) were assembled using Geneious Prime (Biomatters, Auckland, NZ). The published plastome from the same genus served as a reference for related plastome assembly, except for *M*. *tenuifolia* and *P*. *spicata*, because of the lack of published plastomes, as shown in **Supplementary Table 3**. The plastome of *Cremastra appendiculata* and *Trichotosia velutina* were selected as references for plastome assembly of *M*. *tenuifolia* and *P*. *spicata*. Illumina sequence data of these six orchid species were mapped to references for plastome assembly.

### 2.5 Chloroplast-derived sequence detection

To detect chloroplast-derived sequences (cp-derived sequences), we performed a BLASTN (e-value < 1e−5) search of the mitochondrial genomes (*D*. *huoshanense*: LC657527-LC657545; *D*. *chrysanthum*: assembled in this study) against the chloroplast genomes (GenBank accession LC193517; PP479726). Sequences exhibiting more than 80% identity and a length exceeding 200 bp were classified as cp-derived sequences (Gandini et al., 2019). These sequences were then annotated using Vector NTI.

### 2.6 Analyses of repetitive content

The mitochondrial genomes were analyzed for repetitive sequences by performing a self-comparison using BLASTN (e-value < 1e−5). Repetitive sequences showing sequence similarity of over 95% and a minimum length of 20 bp were subsequently identified and quantified. These identified repetitive sequences were categorized into three size classes: short repeats (< 100bp), intermediate repeats (100-1,000bp), and large repeats (>1,000bp). To ensure accurate quantification, an automated script was employed to eliminate any overlap between repeat pairs. To examine recombination frequencies of repeats, we extracted ±2kb single-copy flanking regions of repeat pairs in *D*. *chrysanthum*, built alternative configurations, and mapped corrected long reads to these configurations using minimap2 (https://github.com/lh3/minimap2). Then, we calculated recombination frequency as reads supporting alternative configuration divided by total mapped reads (Wang et al., 2023).

Simple sequence repeats (SSRs) within the mitochondrial genomes were identified using the MISA tool (https://webblast.ipk-gatersleben.de/misa/). The search parameters were configured to detect polynucleotide motifs with a minimum repeat length of five, while mononucleotide motifs were set to a threshold of eight.

### 2.7 Comparison analyses of mitogenomes

For comparative analysis, we retrieved eight published orchid mitogenomes from the NCBI database (https://www.ncbi.nlm.nih.gov/), including two *Dendrobium* species (LC657527-LC657545; LC640134-LC640155), *Cymbidium ensifolium* (OR754281-OR754263), *Phalaenopsis aphrodite* (MN366132-MN366175), *Gastrodia elata* (MF070084-MF070102), *Paphiopedilum micranthum* (OP465200-OP465225), and two *Apostasia* species (PP724664; OQ645347) (Wang et al., 2023; Shen et al., 2024; Chen et al., 2020; Yuan et al., 2018; Yang et al., 2023; Zheng et al., 2024; Ke et al., 2023). The similarities among these mitogenomes were first evaluated using the BLASTN algorithm (https://ftp.ncbi.nlm.nih.gov/blast/executables/blast+/LATEST/), with the e-value <1e−5. The lengths of the detected regions were measured to determine the sequence similarities between each pair of mitogenomes. A custom Python script was used to remove any redundant sequences.

### 2.8 Construction of phylogenetic trees

Mitogenome sequences from 10 orchid species among six recently radiated tribes in Epidendroideae constituted a matrix for mitochondrial phylogeny analysis. To improve the efficiency of mitogenome alignment, each isoform of the mitogenome was aligned respectively using MAFFT v7 (Katoh and Standley, 2013). These aligned isoforms were then concatenated to form the mitochondrial matrix. For plastome phylogeny analysis, we downloaded four published orchid plastomes and assembled six orchid plastomes, constructing a plastome matrix with 10 orchid species (Supplementary **Table 1**). The plastome matrix was also aligned by MAFFT v7. Alignments containing more than 50% missing data were excluded from the analysis. For phylogenetic construction, *Vanilla annamica* was set as the ingroup and *Allium cepa* was set as the outgroup. The Maximum likelihood (ML) trees were resolved based on mitochondrial and chloroplast sequences using RAxML v8.0.0 (Stamatakis, 2014), employing the GTRGAMMA model with 1,000 bootstrap replicates for support estimation. The resulting topologies for the mitochondrial phylogeny were visualized using FigTree v1.4.2 (http://tree.bio.ed.ac.uk/software/figtree/).

**Table 1:** General features of nine orchid mitogenomes.

			Unique gene numbers		
Genome feature	Mitogenome length (bp)	Number of isoforms	Protein-coding genes	tRNA genes	rRNA genes
*Dendrobium huoshanense*	632,571	19	38	17	3
*Dendrobium henanense*	807,551	24	38	19	3
*Dendrobium chrysanthum*	638,041	21	38	20	3
*Phalaenopsis aphrodite*	576,203	44	38	9	3
*Cymbidium ensifolium*	560,647	19	35	36	3
*Gastrodia elata*	1,339,000	19	37	18	3
*Paphiopedilum micranthum*	447,368	26	39	16	3
*Apostasia fujianica*	573,612	1	30	12	2
*Apostasia shenzhenica*	672,872	1	36	16	2

## 3 Results

### 3.1 Mitogenome features of the newly sequenced *Dendrobium* species

**Figure 1:**
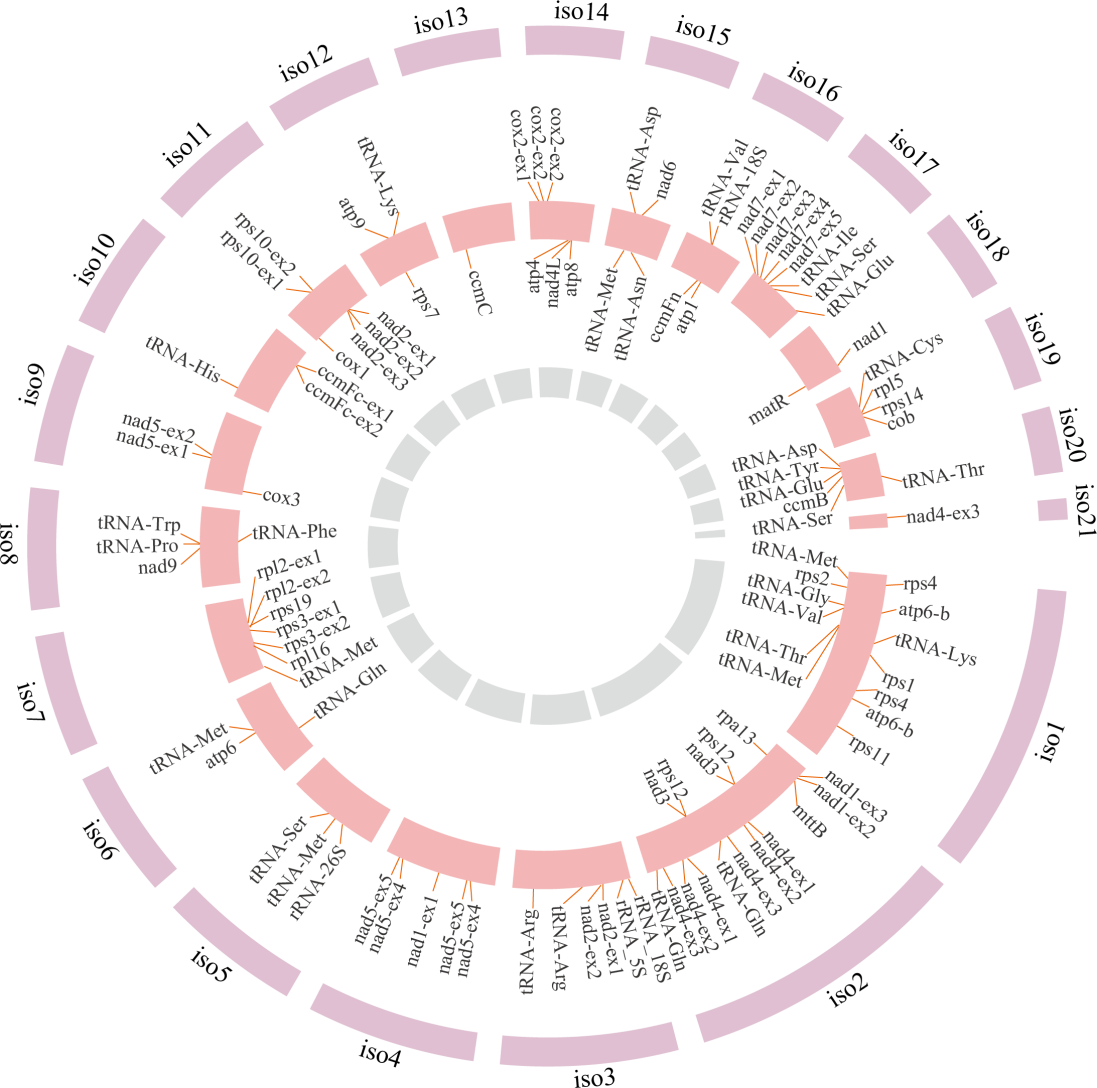
Mitochondrial genome maps of *Dendrobium chrysanthum*. Isoforms of the mitogenome are depicted as a circle; Gene names in the inner ring are reverse genes and gene names in the outer ring are forward genes; “-ex” means exon.

In this study, we characterized the mitochondrial genome of *Dendrobium chrysanthum* (Epidendroideae, Orchidaceae), revealing distinctive multichromosomal architectures (**Figure 1**). The assembled mitogenome was 638,041 bp in total length, comprising 21 isoforms (chromosomes) with individual sizes ranging from 70,862 bp to 5,115 bp (**Supplementary Table 4**). Besides, 14/21 of isoforms exhibited circular configurations ranging from 19,261 bp to 41,234 bp, lacking a conserved ‘master circle’ structure. GC content heterogeneity was observed across isoforms. Gene annotation identified 61 single-copy functional genes, including 38 conserved protein-coding genes, 20 tRNA genes, and 3 rRNA genes. Additionally, a total of 10 protein-coding genes contained cis-spliced introns (*nad4*, *nad7*, *cox2*, *ccmFc*, *rpl2*, *rps3*, and *rps10*) or trans-spliced (*nad1*, *nad2*, and *nad5*). Shared gene losses (*rpl10*, *sdh3*, *sdh4*) mirrored patterns in other orchid species.

### 3.2 Gene clusters

We examined the mitochondrial genome of *D*. *chrysanthum* for the presence of gene clusters, which we defined as two or more adjacent genes comprising a gene cluster (**Supplementary Figure 1**). We found 13 such gene clusters in *D*. *chrysanthum* mitogenomes, including *rrn26*-*trnM-CAT*, *atp8*-*nad4L*-*atp4*, *trnP-TGG*-*trnW-CCA*, *trnE-TTC*-*trnY-GTA*, *atp6*_b-*trnV-TAC*, *rps3*-*rpl16*-*rpl2*-*rps19*, *atp9*-*rps7*, *atp6*-*trnM-CAT*, *atp1*-*ccmFn*, *rrn5*-*rrn18*, *nad7*-*trnI-TAT*, *rps14*-*rpl5*, *nad3*-*rps12*. A comparison among six *Dendrobium* species revealed 16 conserved gene clusters in these mitogenomes. Notably, the *nad9-trnF-GAA*, *nad2-trnY-GTA*, and *trnM-CAT-trnG-GCC* gene clusters were absent in the mitochondrial genome of *D*. *chrysanthum*, as shown in **Supplementary Figure 1**. Despite frequent recombination in angiosperm mitochondrial genomes, some conserved gene clusters were still preserved among angiosperm species. In the *D. chrysanthum* mitogenome, five conserved gene clusters were identified, including *nad4L-atp4, rps3-rpl16-rpl2-rps19, rrn5-rrn18, rps14-rpl5*, and *nad3-rps12*, which are shared with most angiosperm mitochondrial genomes.

### 3.3 Repetitive contents

**Figure 2:**
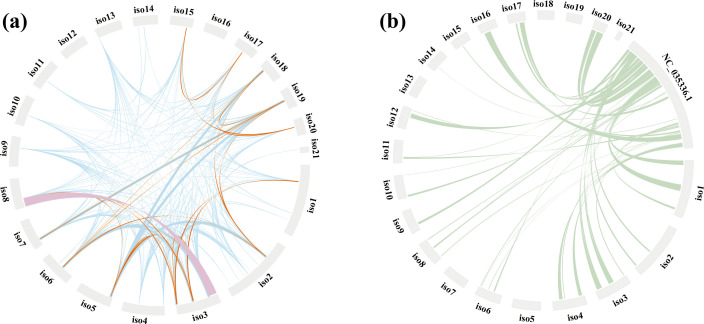
Repetitive sequences and chloroplast-derived sequences of the *D*. *chrysanthum* mitogenome. **(a)** Distribution of repetitive sequences among 21 isoforms of the mitogenome; **(b)** Chloroplast-derived sequences between the chloroplast genome and the mitochondrial genome.

Repetitive sequences play a crucial role in driving sequence rearrangements and structural variations in plant mitochondrial genomes. In this study, a total of 99,862 bp repetitive sequences were identified in the mitochondrial genome of *D*. *chrysanthum*, which were categorized as large (>1,000 bp), intermediate (100-1,000 bp), and short repeats (<100 bp) (**Figure 2**). The number of short repetitive sequences was the largest (406 repeats), followed by intermediate repetitive sequences (288 repeat pairs), and then large repetitive sequences (5 repeats). All detected repetitive sequences accounted for 15.7% (99,862 bp) of the length of the whole *D*. *chrysanthum* mitogenome. The large repeats contributed the greatest cumulative length (84,330 bp), followed by intermediate (11,609 bp) and short repeats (3,923 bp). Most repeat pairs are distributed between isoforms. In addition, we calculated recombination frequencies for each repeat pair using long-read sequencing data (**Supplementary Figure 2**). Seven repeat pairs, with lengths ranging from 200 bp to 900 bp, exhibited recombination events. The corresponding recombination frequencies ranged from 0.01 to 0.17.

Simple sequence repeats (SSRs) serve as valuable molecular markers for investigating plant population genetics, evolutionary biology, and ecological dynamics. Here, simple sequence repeats were identified and quantified in the mitochondrial genomes of *D*. *chrysanthum* (**Supplementary Table 4**). Only mononucleotide and dinucleotide repeats were detected across the mitogenome. A total of 37 SSRs were distributed among 21 isoforms of the *D*. *chrysanthum* mitogenome. The distribution of these SSRs showed extreme inconsistency across mitochondrial isoforms. The isoform 20 contained the greatest number of simple sequence repeats (21 SSRs), while the isoform 2, isoform 3, isoform 5, isoform 8, isoform 9, isoform 10, isoform 12, isoform 17, and isoform 21 only contained 1–5 SSRs, respectively. More specifically, more than half of all isoforms lacked SSRs entirely. Correlation analyses revealed non-significant associations between isoform length and SSR density in *D*. *chrysanthum* (Pearson’s r=-0.14, P > 0.05). To further investigate potential drivers of this uneven distribution, GC content was analyzed for correlations with SSR abundance. The GC content also showed non-significant correlations with SSR density (Pearson’s r=-0.29, P > 0.05).

### 3.4 Chloroplast-derived sequences

We detected 94,765 bp chloroplast-derived (cp-derived) sequences in the mitogenomes of *D*. *chrysanthum*, representing 14.9% of the mitochondrial genome lengths. The cp-derived sequences of *D*. *chrysanthum* mitogenome ranged from 205 to 4,392 bp (**Supplementary Figure 3**). Among them, sequences of 200–500 bp were most frequent (31 sequences), followed by 501-1,000 bp (16 sequences) and 1001-2,000 bp (16 sequences).

To analyze distribution patterns, cp-derived sequences were mapped across mitochondrial isoforms (**Figure 2**; **Supplementary Table 5**). These sequences displayed inconsistent localization across isoforms, independent of isoform length. Excluding isoform 5, isoform 7, isoform 13, isoform 18, isoform 19, and isoform 21, the rest of the isoforms contained cp-derived sequences with the number ranging from 1 to 16. Additionally, the isoform 20 (15,980 bp) with the most cp-derived sequences was not the longest one among the 21 isoforms in the *D*. *chrysanthum* mitogenome. The correlation among cp-derived sequences and isoform length also showed low correlation between these two indicators (Pearson’s r=0.3, P > 0.05).

### 3.5 Mitogenome comparison of three *Dendrobium* species and six other orchid species

We compared nine mitochondrial genomes of three *Dendrobium* species and six other orchid species from three orchid subfamilies, including six species of Epidendroideae, a species of Cypripedioideae, and two species of Apostasioideae (**Table 1**). Except for the mitochondrial genome of *Dendrobium chrysanthum*, the other eight mitochondrial genomes were downloaded from the NCBI (https://www.ncbi.nlm.nih.gov/). Significant expansion characterizes the evolution of mitochondrial genome size within Orchidaceae, ranging from 1,339,000 bp (*Gastrodia elata*) to 447,368 bp (*Paphiopedilum micranthum*). The length of the *G*. *elata* mitogenome was approximately three times that of the mitogenome of *P*. *micranthum*. Such differences were not only found among different subfamilies, but also existed in the same genus, e.g., the mitogenome size of *Dendrobium henanense* and *Dendrobium huoshanense*. Furthermore, most examined mitochondrial genomes of these orchid species feature multi-chromosomal architectures. The mitochondrial genome of *Phalaenopsis aphrodite* consisted mostly of multi-chromosomal architectures (44 isoforms), while the *Apostasia fujianica* mitogenome and the *Apostasia shenzhenica* mitogenome only showed a circular genomic draft. The variability of mitogenome structure was a general characteristic in orchid species, reflecting their rapid structural evolution.

**Figure 3:**
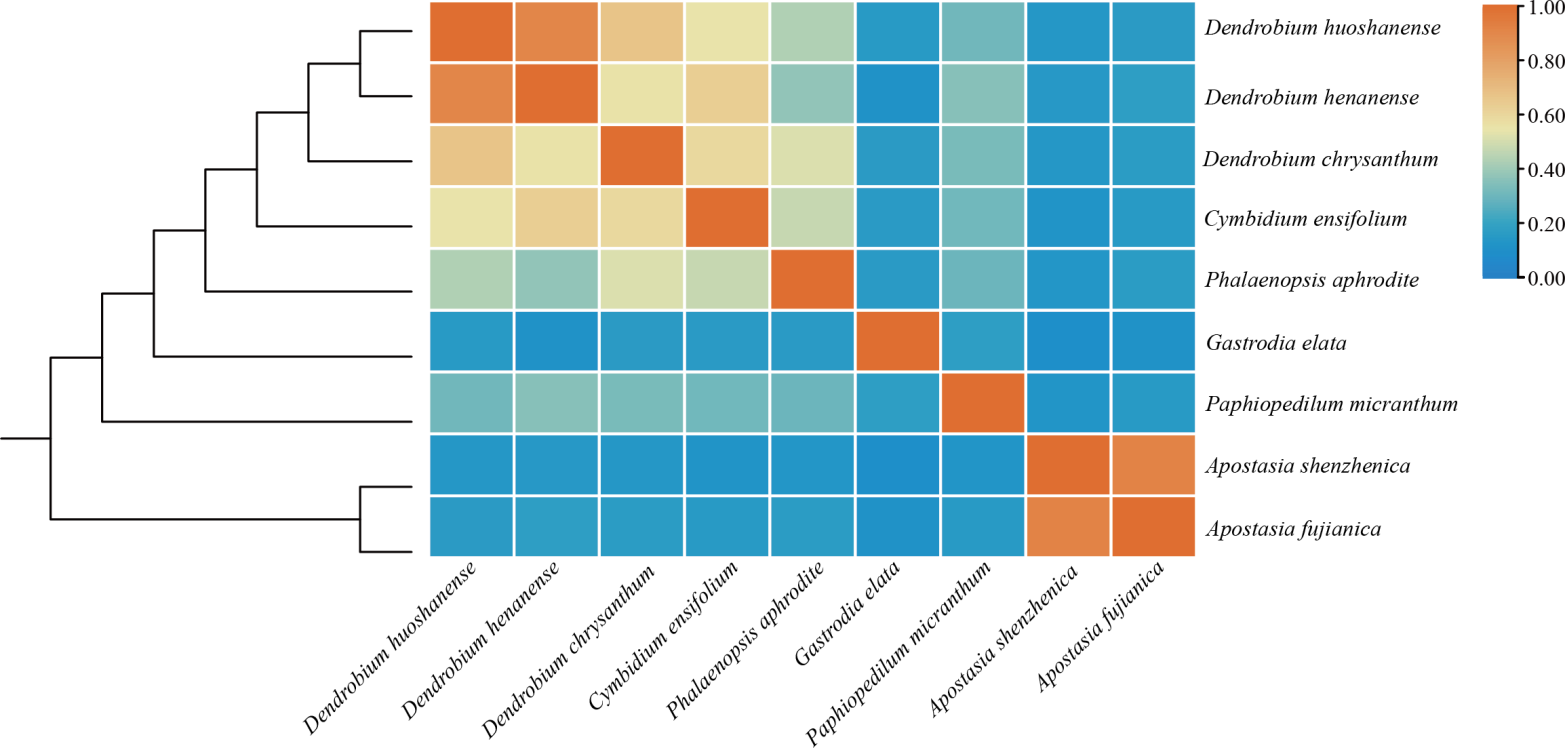
Genomic similarities among Orchidaceae mitogenomes. Similarity among nine Orchidaceae mitogenomes. Blue represents low similarity. Red represents relatively high similarity.

Sequence similarities among these nine orchid mitogenomes were detected, which revealed that the content of shared sequences closely related to the phylogenetic affinity (**Figure 3**). Species from the same genus shared more mitogenome sequences than other orchid species, e.g., mitogenomes of *Apostasia shenzhenica* and *Apostasia fujianica* shared approximately 92% of sequences, as well as the *Dendrobium* genus. Mitogenome sequences were indicated to be relatively conserved at the genus level. Although *Phalaenopsis Aphrodite* and *Cymbidium ensifolium* were both from Epidendroideae, their mitogenomes only had 47% of sequence similarity. It should be noted that the mitochondrial genome of *Gastrodia elata* was an exception here. Sequence similarities between the *G*. *elata* mitogenome and other species from Epidendroideae were less than 16%, yet the *Paphiopedilum micranthum* mitogenome shared more than 30% of sequences with other Epidendroideae species. It is probably related to the parasitic characteristic of *G*. *elata*.

### 3.6 Phylogenetic analyses

**Figure 4:**
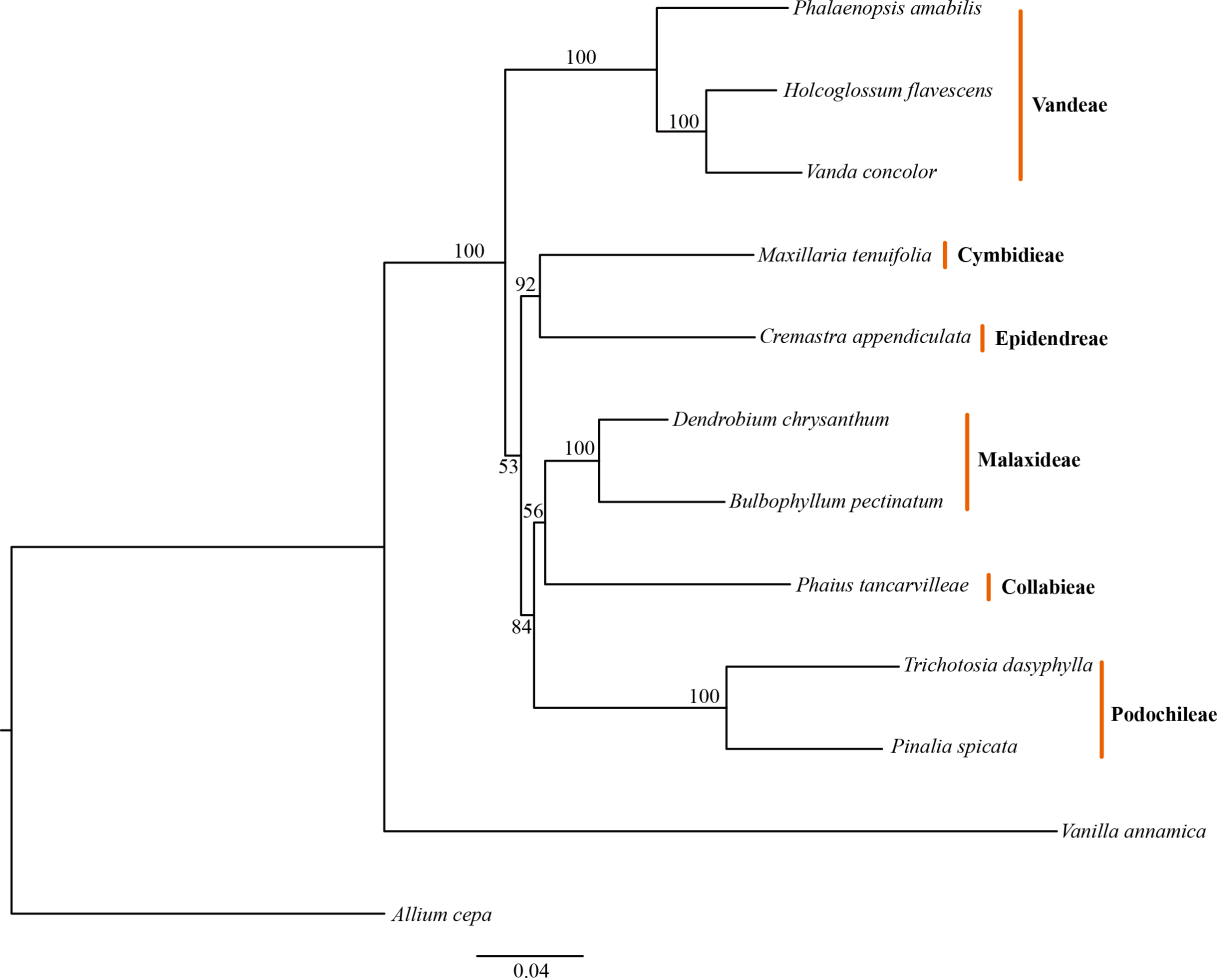
The phylogeny of 10 individuals from six recently radiated tribes in Epidendroideae based on complete mitogenomes. The number on each branch was the bootstrap support (BS) of the ML analysis. Only BS > 50% are shown near the nodes.

**Figure 5:**
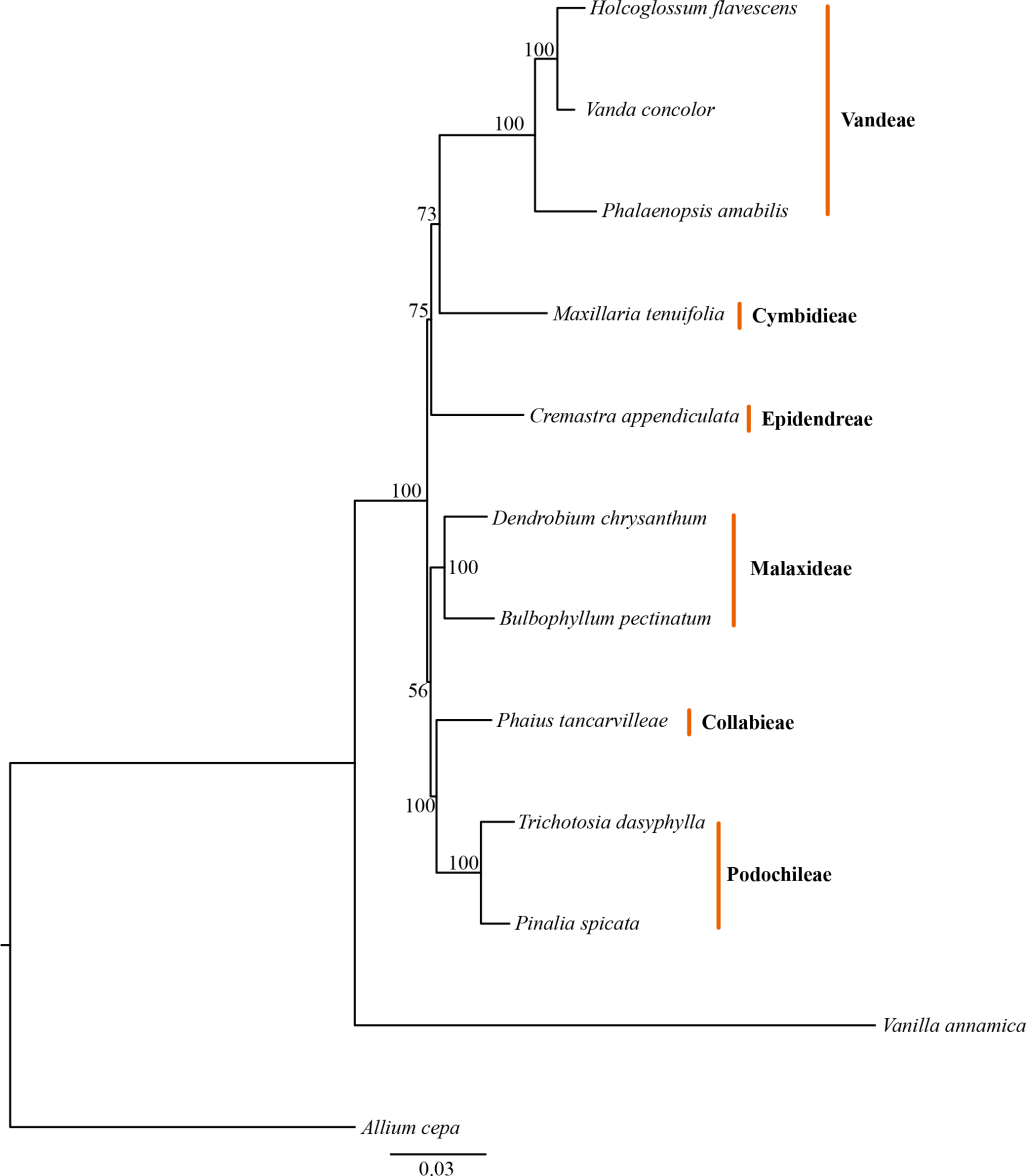
The phylogeny of 10 individuals from six recently radiated tribes in Epidendroideae based on complete chloroplast genomes. The number on each branch was the bootstrap support (BS) of the ML analysis. Only BS > 50% are shown near the nodes.

Mitochondrial genomes of 10 orchid species were newly assembled, with the *D*. *huoshanense* mitogenome as a reference. Chloroplast genomes of six orchid species were also newly assembled in this study. We reconstructed phylogenetic relationships of six tribes in Epidendroideae based on complete mitochondrial genomes and chloroplast genomes (**Figures 4**, **5**). Both mitochondrial and plastome trees recovered a monophyletic group of the six tribes in Epidendroideae. Moreover, the backbone of mitochondrial and plastome trees was strongly supported (bootstrap supports>80%) except for the two nodes.

The mitochondrial tree showed that the Malaxideae clade and Collabieae clade were clustered into a monophyletic group. The Podochileae tribe was sister to Malaxideae and Collabieae with high support. The Cymbidieae and Epidendreae tribes were sister clades with 92% bootstrap support. The Vandeae tribe was successively sister to the other five tribes in Epidendroideae with 100% bootstrap support.

Topologies of the mitochondrial tree are generally congruent with the plastid tree, but with exceptions in some tribes. Bootstrap support of some nodes was also different. For instance, Cymbidieae and Epidendreae were paraphyly, while these two tribes were clustered into a monophyly in the mitochondrial tree. Moreover, Collabieae was the sister clade of Podochileae with 100% bootstrap support, which was heterogenetic with the phylogenetic position in the mitochondrial tree.

## 4 Discussion

### 4.1 Conserved and variable evolutionary features across orchid mitogenomes

As one of the most species-rich families in angiosperms, Orchidaceae diverged at more than 100 million years ago (Ma), and the five subfamilies diverged from each other at 67Ma-86Ma (Li et al., 2019). Here, nine mitochondrial genomes from three Orchidaceae subfamilies were compared to assess their evolutionary features. Until now, 41 protein-coding genes have been identified in angiosperm mitogenomes (Richardson et al., 2013). These orchid mitogenomes present 30–39 protein-coding genes (Wang et al., 2023; Shen et al., 2024; Chen et al., 2020; Yuan et al., 2018; Yang et al., 2023; Zheng et al., 2024; Ke et al., 2023). Partial protein-coding genes were missed during evolution. Of these missing genes, the *rpl10* and *sdh3* were lost from all analyzed orchid mitogenomes. Less common is the gene loss event in the *Apostasia fujianica* mitogenome that lost approximately 1/4 protein-coding genes compared with other orchid species. The frequent transfer of mitochondrial genes to the nuclear genome, followed by functional activation of the nuclear copies, leads to the redundancy and eventual loss of the original mitochondrial genes (Garcia et al., 2021; Warren et al., 2021, 2023). Moreover, the pattern of such gene loss is episodic in plant mitochondrial genomes (Adams and Palmer, 2003).

Genome sizes varied among these orchid mitogenomes. Most orchid mitogenomes were 550kb-650kb long. The genome size of the *Paphiopedilum micranthum* mitogenome was 447kb, which was the smallest mitogenome among orchid species. The mitogenome sizes of *Gastrodia elata* and *Dendrobium henanense* were larger than all analyzed orchid species, with 1,339kb and 807kb, respectively. Such significant variation in mitogenome sizes was also found in other plant families. In Solanaceae, the genomic size varied from 424kb to 685kb based on the comparison of six mitochondrial genomes from five genera (Gandini et al., 2019). As for the entire angiosperms, mitogenome sizes have undergone inconceivable changes, with the range from 208kb (*Brassica hirta*) to 11.3Mb (*Silene conica*) (Skippington et al., 2015; Sloan et al., 2012).

Mitochondrial genomes of flowering plants are well-known for their extreme structural variations, which are related to recombination activities mediated by repetitive sequences (Kozik et al., 2019; Cole et al., 2018). Benefiting from the maturation of third-generation sequencing technology, complex structures of plant mitogenomes were deciphered in many lineages (Yang et al., 2025; Luo et al., 2025; Ye et al., 2025; Cai et al., 2025; Liu et al., 2025). Mitochondrial genomes of nine orchid species also showed obvious structural variations during evolution. In Epidendroideae, all mitochondrial genomes displayed a multi-chromosomal architecture rather than one genome-sized circle. Among them, the mitochondrial genome of *Phalaenopsis Aphrodite* comprised the most sub-genomes (44 isoforms), followed by *Dendrobium henanense mitogenome* with 24 isoforms. Most isoforms of these mitogenomes formed a closed loop that distributed several mitochondrial genes. In fact, previous studies have proved that plant mtDNAs were unequally distributed in different mitochondria, and a part of mitochondria only contained an incomplete mitogenome, which was related to the multi-chromosomal structure of plant mitogenomes (Arimura, 2018). Nevertheless, mitochondria with incomplete mitogenomes still could maintain functions through frequent fusion and fission for genetic material sharing (Sheahan et al., 2005; Arimura et al., 2004).

Mitochondrial sequence similarity analysis of nine orchid species revealed that the closer phylogenetic relationship, the more sequences are shared between these mitogenomes. For instance, the *Dendrobium huoshanense* mitogenome shared more than 90% sequence with the mitogenome of *Dendrobium henanense*, as the closely related species in the *Dendrobium* genus. The same situation was also reported in other plant lineages. The study of Gandini et al. (2019) compared five Solanaceae mitogenomes, revealing a relatively high sequence similarity between two closely related species. However, the *Gastrodia elata* mitogenome was an exception due to its special parasitic characteristic. Although the phylogenetic position of *Gastrodia elata* was closer to *Phalaenopsis Aphrodite* than *Paphiopedilum micranthum*, the *P*. *Aphrodite* mitogenome still shared more sequences with the *P*. *micranthum* mitogenome rather than the mitogenome of *G*. *elata*. Generally, sequence similarity was a simple and comprehensible characteristic compared to the size and the structure of plant mitogenomes, which could reflect phylogenetic affinity under most circumstances.

### 4.2 Mitochondrial sequences resolving the interrelationships of Epidendroideae (Orchidaceae)

The Orchidaceae lineage, characterized by exceptional species radiation and divergent morphological traits, constitutes a model system for addressing fundamental questions in plant phylogenetics (Lin et al., 2015; Sabino Kikuchi et al., 2020; Thompson et al., 2023). Molecular systematics has yielded a comprehensive phylogenetic framework for Orchidaceae that is subdivided into five subfamilies, including Epidendroideae, Orchidoideae, Apostasioideae, Cypripedioideae, and Vanilloideae (Cameron et al., 1999; Chase et al., 2003). However, phylogeny relationships in these subfamilies still persist in unresolved clades, especially in Epidendroideae. Most conflict clades of Orchidaceae were in Epidendroideae based on research since 2015 (Wang et al., 2024). Previous studies supported that Epidendroideae consisted of 16 tribes (Zhang et al., 2023; Givnish et al., 2015). Although positions of most tribes were stable, there remained confusion about phylogenetic relationships of several recently radiated tribes (Kim et al., 2020; Kim and Kim, 2022; 14, Pérez-Escobar et al., 2024; Zhang et al., 2023). For example, the phylogenetic relationships of Vandeae, Epidendreae, and Cymbidieae were controversial among different studies. There were two perspectives: (1) The phylogenies based on nuclear and plastid markers always supported that Vandeae and Cymbidieae clustered into a monophyletic group, and Epidendreae was sister to them (Serna-Sánchez et al., 2021; Givnish et al., 2015; Wong and Peakall, 2022); (2) Another perspective adopted that Epidendreae and Cymbidieae were clustered into a monophyly, and Vandeae was sister to them (Li et al., 2019; Pérez-Escobar et al., 2024; Zhang et al., 2023; Freudenstein and Chase, 2015; Pérez-Escobar et al., 2021). In this study, we reconstructed phylogenetic relationships of six tribes in Epidendroideae based on complete mitochondrial genomes and chloroplast genomes. Most controversial clades in Epidendroideae were resolved, especially for the mitochondrial phylogeny that provided new insights into the interrelationships of Epidendroideae. The comparative analysis of phylogenetic topology showed discordances between the plastid tree and the mitochondrial tree. For the three controversial tribes in Epidendroideae (Vandeae, Epidendreae, and Cymbidieae), the plastid phylogeny supported the first perspective as mentioned in the previous text, while the phylogenetic relationship of these three tribes was consistent with the second perspective. Besides, the position of Collabieae and Podochileae also showed conflict between the plastid tree and the mitochondrial tree in our study. Collabieae was the sister clade of Podochileae with 100% bootstrap support in the plastid phylogeny, which was heterogenetic with the phylogenetic position in the mitochondrial tree. Paternal leakage and substitution rate heterogeneity of mitogenomes or plastomes were potential factors for such phylogenetic conflict (Noyszewski et al., 2014; Hénault et al., 2022; Liu et al., 2014). The phylogenetic conflict between plastid and mitochondrial trees was a universal phenomenon that has been reported by many phylogenetic analyses (Kapli et al., 2021; Postel et al., 2024; Mandel et al., 2020). In Orchidaceae, the conflict was found between plastid and mitochondrial trees based on 78 plastid and 38 mitochondrial protein coding sequences, and different substitution rates and RNA editing of plastid and mitochondrial sequences were the potential causes (Wang et al., 2022; Lukeš et al., 2021; Khachaturyan et al., 2024). Because of different sampling strategies and datasets, coupled with practical limitations, it is still a challenge that reconstruct more robust and reliable phylogenies of orchids. Some controversial and unresolved clades in Orchidaceae needed a more effective approach to decipher these complex evolutionary relationships.

## 5 Conclusion

In this study, we *de novo* assembled the complete mitogenomes of *Dendrobium chrysanthum* that was 638,041 bp long with a multichromosomal structure. The comparative analysis of nine mitogenomes from three orchid subfamilies showed great variations in the structure and genome size, while sequence similarities among these mitogenomes were closely related to the phylogenetic affinity. We also newly assembled ten mitochondrial genomes, and complete mitochondrial sequences were used to reconstruct the phylogeny of ten orchid species from the six recently radiated tribes of Epidendroideae. Moreover, the plastid phylogeny based on whole chloroplast sequences was reconstructed for these tribes. Most clades were resolved in both the mitochondrial and plastid phylogeny. Notably, plastid and mitochondrial phylogenies displayed discordances in the phylogenetic topologies. Our findings enriched the mitogenome database and provided new insights into the interrelationships of Epidendroideae (Orchidaceae).

## Data Availability

The datasets presented in this study can be found in online repositories. The names of the repository/repositories and accession number(s) can be found below: https://www.ncbi.nlm.nih.gov/genbank/, PRJNA933365.

## Supplementary material

Supplementary Figure 1 Gene clusters of six *Dendrobium* mitogenomes.

Supplementary Figure 2 Numbers of cp-derived sequences of different lengths.

Supplementary Figure 3 Repeat-mediated recombination frequencies of *D*. *chrysanthum* mitogenome.
