# Supplementary figures and images for: The multichromosomal structure evolution of Dendrobium mitogenomes and new insights into interrelationships of recently radiated tribes in Epidendroideae (Orchidaceae)
Source: Front Plant Sci. 2026 Jun 5;17:1864920. doi: 10.3389/fpls.2026.1864920 (PMC13279703; doi:10.3389/fpls.2026.1864920)

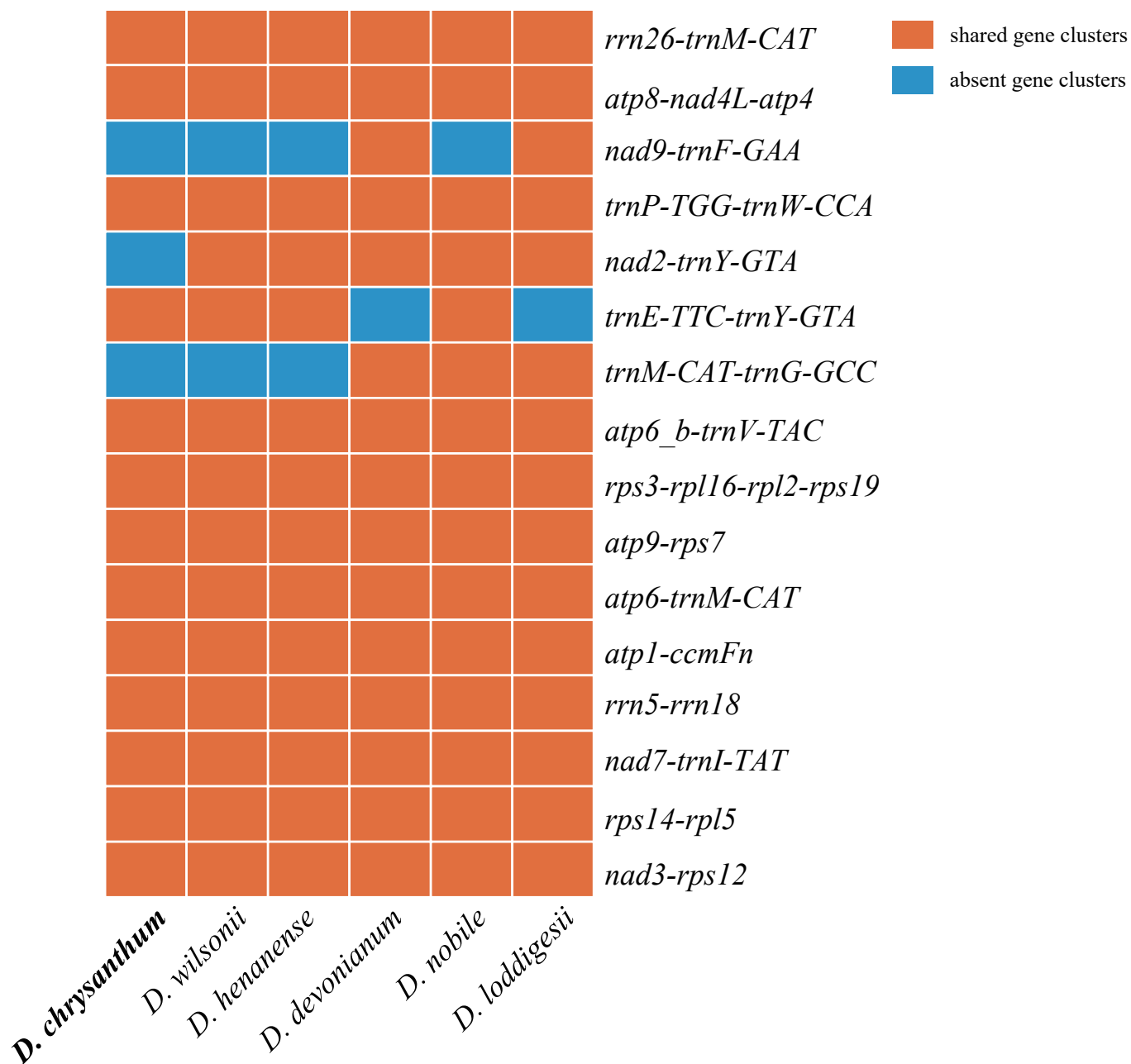

Supplement: Supplementary Figure 1 — Gene clusters of six Dendrobium mitogenomes. [file DataSheet1.pdf]

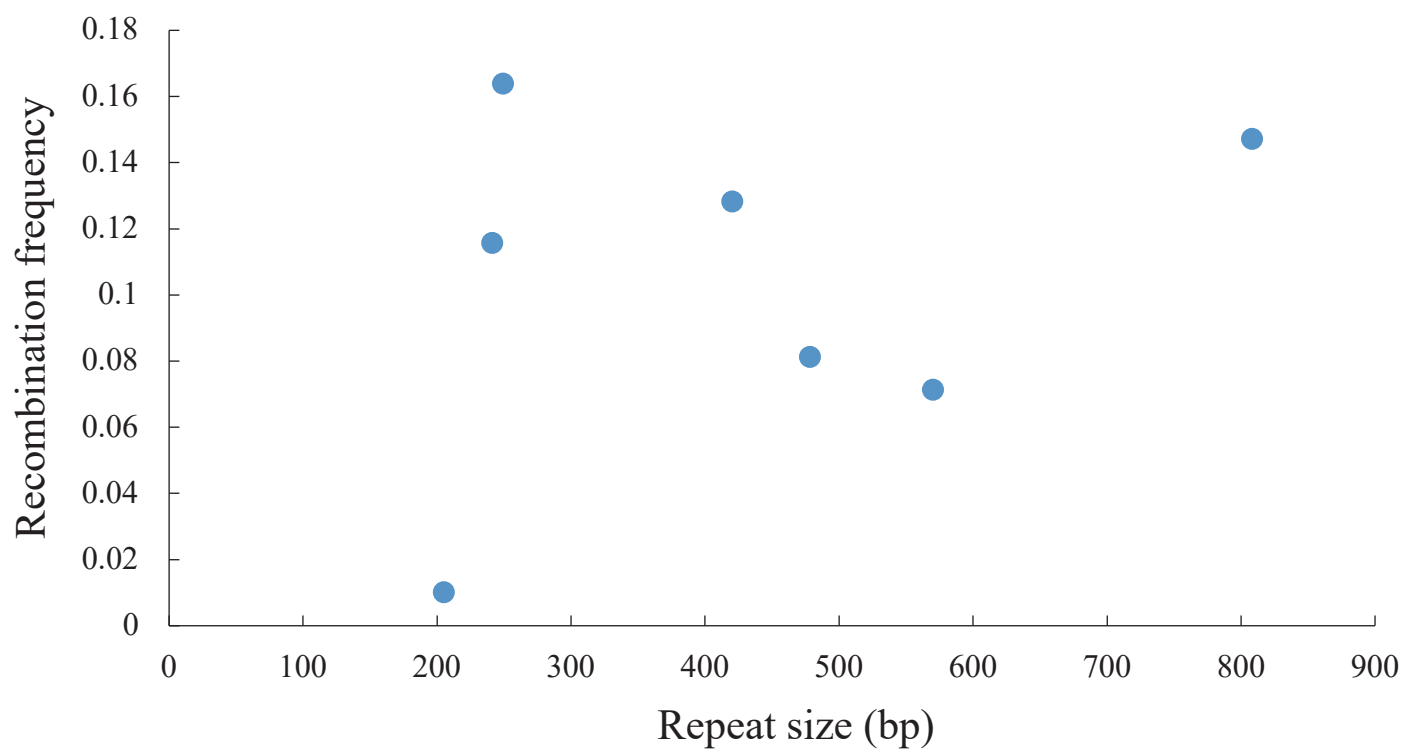

Supplement: Supplementary Figure 2 — Numbers of cp-derived sequences of different lengths. [file DataSheet2.pdf]

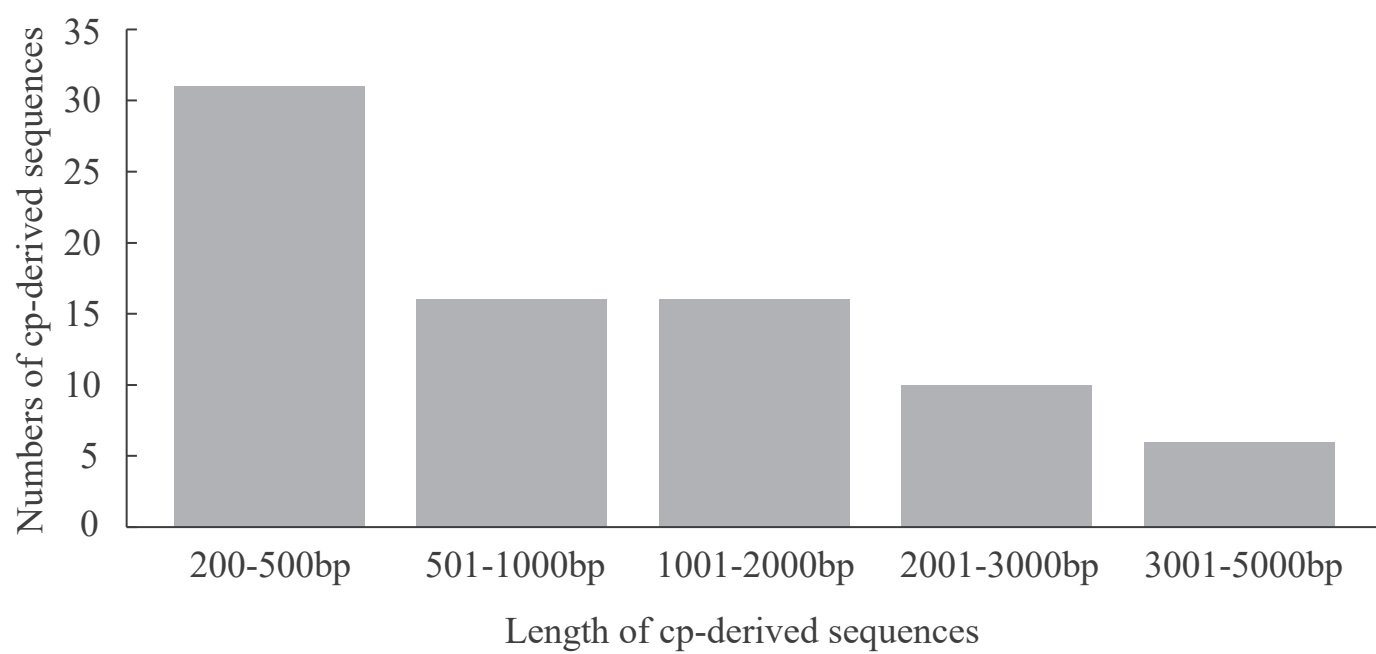

Supplement: Supplementary Figure 3 — Repeat-mediated recombination frequencies of D. chrysanthum mitogenome. [file DataSheet3.pdf]
