# Supplementary material for: The multichromosomal structure evolution of Dendrobium mitogenomes and new insights into interrelationships of recently radiated tribes in Epidendroideae (Orchidaceae)
Source: Front Plant Sci. 2026 Jun 5;17:1864920. doi: 10.3389/fpls.2026.1864920 (PMC13279703; doi:10.3389/fpls.2026.1864920)
Supplement: Supplementary file 4 [file Table1.docx]

| Tribes | Genus | Species |
| --- | --- | --- |
| Vandeae | *Phalaenopsis* | *Phalaenopsis amabilis* |
|  | *Holcoglossum* | *Holcoglossum flavescens* |
|  | *Vanda* | *Vanda concolor* |
| Collabieae | *Phaius* | *Phaius tancarvilleae* |
| Cymbidieae | *Maxillaria* | *Maxillaria tenuifolia* |
| Epidendreae | *Cremastra* | *Cremastra appendiculata* |
| Podochileae | *Trichotosia* | *Trichotosia dasyphylla* |
|  | *Pinalia* | *Pinalia spicata* |
| Malaxideae | *Dendrobium* | *Dendrobium chrysanthum* |
|  | *Bulbophyllum* | *Bulbophyllum pectinatum* |

Table S1. Ten orchid species samples from six tribes in Epidendroideae
