# Supplementary material for: The multichromosomal structure evolution of Dendrobium mitogenomes and new insights into interrelationships of recently radiated tribes in Epidendroideae (Orchidaceae)
Source: Front Plant Sci. 2026 Jun 5;17:1864920. doi: 10.3389/fpls.2026.1864920 (PMC13279703; doi:10.3389/fpls.2026.1864920)
Supplement: Supplementary file 6 [file Table3.docx]

Table S3. References for the assembly of six plastome

| Numbers | Plastome references | Assembled plastomes |
| --- | --- | --- |
| 1 | *Phalaenopsis aphrodite* (NC_007499) | *Phalaenopsis amabilis* |
| 2 | *Holcoglossum kimballianum* (MZ681477) | *Holcoglossum flavescens* |
| 3 | *Cremastra appendiculata* (NC_037439) | *Maxillaria tenuifolia* |
| 4 | *Trichotosia velutina* (OR544616) | *Trichotosia dasyphylla* |
| 5 | *Trichotosia velutina* (OR544616) | *Pinalia spicata* |
| 6 | *Vanilla somae* (NC_079955) | *Vanilla annamica* |
