# Supplementary material for: The multichromosomal structure evolution of Dendrobium mitogenomes and new insights into interrelationships of recently radiated tribes in Epidendroideae (Orchidaceae)
Source: Front Plant Sci. 2026 Jun 5;17:1864920. doi: 10.3389/fpls.2026.1864920 (PMC13279703; doi:10.3389/fpls.2026.1864920)
Supplement: Supplementary file 7 [file Table4.docx]

| Isoform | Form | Length | | GC% content | | Numbers of SSRs |
| --- | --- | --- | --- | --- | --- | --- |
| isoform1 | Linear | 70857 | 0.4143 | | 0 | |
| isoform2 | Linear | 69571 | 0.4609 | | 2 | |
| isoform3 | Linear | 43154 | 0.416 | | 5 | |
| isoform4 | Circular | 41234 | 0.4262 | | 0 | |
| isoform5 | Circular | 34633 | 0.4502 | | 1 | |
| isoform6 | Linear | 31626 | 0.4246 | | 0 | |
| isoform7 | Circular | 31311 | 0.4173 | | 0 | |
| isoform8 | Circular | 29684 | 0.4567 | | 3 | |
| isoform9 | Circular | 28979 | 0.4468 | | 1 | |
| isoform10 | Circular | 28979 | 0.4349 | | 1 | |
| isoform11 | Circular | 27838 | 0.4491 | | 0 | |
| isoform12 | Circular | 26559 | 0.4227 | | 1 | |
| isoform13 | Circular | 25553 | 0.4419 | | 0 | |
| isoform14 | Circular | 23882 | 0.4478 | | 0 | |
| isoform15 | Linear | 22150 | 0.4357 | | 0 | |
| isoform16 | Circular | 22058 | 0.4667 | | 0 | |
| isoform17 | Circular | 21511 | 0.4296 | | 1 | |
| isoform18 | Circular | 20173 | 0.4532 | | 0 | |
| isoform19 | Circular | 19261 | 0.4434 | | 0 | |
| isoform20 | Linear | 15980 | 0.3712 | | 21 | |
| isoform21 | Linear | 5115 | 0.2545 | | 1 | |

Table S4. Isoform features of *Dendrobium chrysanthum* mitogenome
