# Supplementary material for: The multichromosomal structure evolution of Dendrobium mitogenomes and new insights into interrelationships of recently radiated tribes in Epidendroideae (Orchidaceae)
Source: Front Plant Sci. 2026 Jun 5;17:1864920. doi: 10.3389/fpls.2026.1864920 (PMC13279703; doi:10.3389/fpls.2026.1864920)
Supplement: Supplementary file 8 [file Table5.docx]

Table S5. Distribution patterns of cp-derived sequences among the *D*. *chrysanthum* mitogenome.

| Isoform | Number of cp-derived sequences |
| --- | --- |
| isoform1 | 13 |
| isoform2 | 6 |
| isoform3 | 9 |
| isoform4 | 4 |
| isoform6 | 4 |
| isoform8 | 4 |
| isoform9 | 1 |
| isoform10 | 2 |
| isoform11 | 3 |
| isoform12 | 4 |
| isoform14 | 1 |
| isoform15 | 2 |
| isoform16 | 4 |
| isoform17 | 6 |
| isoform20 | 16 |
